# Rapid and Easy Detection of Microcystin-LR Using a Bioactivated Multi-Walled Carbon Nanotube-Based Field-Effect Transistor Sensor

**DOI:** 10.3390/bios14010037

**Published:** 2024-01-11

**Authors:** Myeongsoon Lee, Seong H. Kim, Don Kim, Hak Jun Kim

**Affiliations:** 1 Department of Chemistry, Pukyong National University, Busan 48513, Republic of Korea; mhleen@gmail.com (M.L.); donkim@pknu.ac.kr (D.K.); 2 Department of Chemical Engineering and Materials Research Institute, Pennsylvania State University, University Park, PA 16802, USA; shk10@psu.edu

**Keywords:** DNA aptamer, FET sensor, cyanotoxins, carbon nanotube, microcystin-LR

## Abstract

In this study, we developed a multi-walled carbon nanotube (MWCNT)-based field-effect transistor (MWCNT-FET) sensor with high sensitivity and selectivity for microcystin-LR (MC-LR). Carboxylated MWCNTs were activated with an MC-LR-targeting aptamer (MCTA). Subsequently the bioactivated MWCNTs were immobilized between interdigitated drain (D) and source (S) electrodes through self-assembly. The top-gated MWCNT-FET sensor was configured by dropping the sample solution onto the D and S electrodes and immersing a Ag/AgCl electrode in the sample solution as a gate (G) electrode. We believe that the FET sensor’s conduction path arises from the interplay between the MCTAs, with the applied gate potential modulating this path. Using standard instruments and a personal computer, the sensor’s response was detected in real-time within a 10 min time frame. This label-free FET sensor demonstrated an impressive detection capability for MC-LR in the concentration range of 0.1–0.5 ng/mL, exhibiting a lower detection limit of 0.11 ng/mL. Additionally, the MWCNT-FET sensor displayed consistent reproducibility, a robust selectivity for MC-LR over its congeners, and minimal matrix interferences. Given these attributes, this easily mass-producible FET sensor is a promising tool for rapid, straightforward, and sensitive MC-LR detection in freshwater environments.

## 1. Introduction

Warm weather, eutrophication, and excessive nutrient richness can trigger cyanobacterial outbreaks in freshwater systems. Cyanobacteria produce various microcystins (MCs) [1]. One particularly toxic variant is Microcystin-LR (MC-LR), characterized by leucine (L) and arginine (R) located at the second and fourth positions of its five non-proteinogenic amino acids. Its lethal dose (LD_50_) is quantified at 43 μg/kg [2]. Consequently, the World Health Organization (WHO) advises maintaining MC-LR levels in drinking water under 1 μg/L [3].

While high-performance liquid chromatography paired with tandem mass spectrometry (LC/MS/MS) excels as the premier analytical method for MC-LR quantification—boasting a detection limit of ~0.01 ng/mL [4]—its inapplicability for field tests at contaminated sites indicates that water samples often need transport to labs. This limitation underscores the importance of developing field-deployable MC-LR detection methods, particularly for remote areas.

Several antibody-based immunoassay approaches for MC-LR detection have been extensively investigated owing to their notable sensitivity and selectivity. These include enzyme-linked immunosorbent assays (ELISAs), and various types of immunosensors based on optical [5,6], piezoelectric [7], electrochemical signals [8,9,10,11,12,13,14,15], and protein phosphatase inhibition (PPI) assays [16,17,18] with detection limits ranging from as low as 7 pg/L [6] to 1 ng/L [7]. However, these methods are time-consuming and require specialized equipment.

Aptamers, which are synthetic single-stranded RNA or DNA molecules, have drawn significant scientific interest because of their specific interactions with target molecules, analogous to antibodies [19]. DNA aptamers, owing to their ease of synthesis, chemical modifiability, robust stability, and reversible denaturation, are currently prime candidates for biomolecular recognition using biosensing techniques [20]. Notably, an MC-LR-targeting aptamer (MCTA; DNA oligonucleotide, 5-NH_2_-C_6_-AN_6_) was identified from random DNA/RNA sequence pools using the SELEX selection process [9].

The integration of one-dimensional (1-D) nanomaterials into field-effect transistors (FETs) can amplify the detection sensitivity and speed, considering their large surface area and superior physicochemical properties [21,22,23,24]. Single-walled carbon nanotubes (SWCNTs) have emerged as the most promising 1-D material for field-effect transistor (FET) biosensor applications because of their large surface area, high aspect ratio, high conductivity, and good physical stability [22]. The adsorption of biological molecules onto SWCNTs results in a significant electric-field perturbation during electron transport in the CNT owing to the electrostatic gating effect, gate coupling, and changes in carrier mobility [23,24]. Notably, the single-molecule detection of proteins and DNA has been achieved using CNT-based FET biosensors [19,25]. Furthermore, one research group [8] demonstrated modified SWCNTs, which were grown between the drain (D) and source (S) electrodes and subsequently bioactivated with MC-LR antibodies, for use as FET-sensing elements for MC-LR detection.

Multi-walled carbon nanotubes (MWCNTs) traditionally offer a lower efficacy than FET elements because the electrical properties of the internal layer tubes of MWCNTs are not readily modulated by the applied gate field. This intrinsic inefficacy problem can be compensated using extrinsic functionalization methods. MWCNTs modified with metallic nanoparticles (like Pt and Au) were developed by our group as possible active elements for sugar-detecting FET sensors [26,27]. The electrical conduction path in the nanoparticle-attached MWCNTs is assumed to involve contact between the metallic surfaces, which enables the adsorption of organic chemicals.

Based on this finding, we postulate that if the electrical conduction path of *p*-type conducting MCTA-activated MWCNTs involves overlapping connections between immobilized MCTAs, such bioactivated MWCNTs can potentially be employed as excellent active elements in FET devices. The development of a biochemically activated MWCNT-based paper-type immunosensor for assaying prostate-specific antigen (PSA) [28] and MC-LR [29] was explored in our previous studies; the sensor exhibited acceptable low detection limits (1.18 ng/mL for PSA and 0.19 ng/mL for MC-LR). Although this method is straightforward for detecting the target compounds, it requires a long detection time (90 min) and yields a high detection limit comparable to that of LC/MS/MS-based MC-LR detection (0.01 g/mL).

**Scheme 1:**
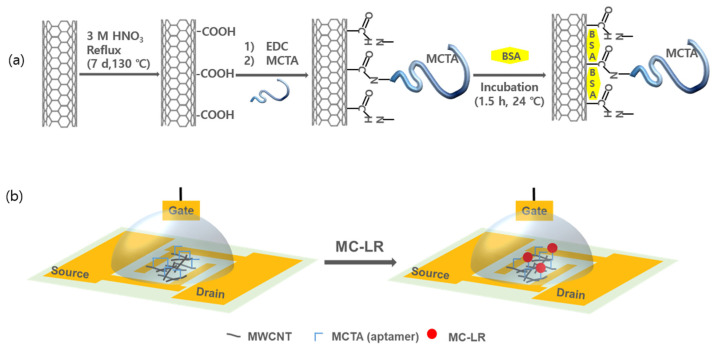
Schematic illustration of top-gated field-effect transistor using functionalized MWCNTs. (**a**) Bioactivation of MWCNTs via chemical bonding between microcystin-LR targeted aptamers (MCTAs) and carboxylated MWCNTs. (**b**) Selective interactions between MC-LR and the MCTAs attached to the top-gated field-effect transistor (FET).

In this study, we demonstrate further advancement of the MC-LR assay using a reliable and rapid-response top-gated FET sensor. The biosensor was assembled using bioactivated MCTA-MWCNTs between two Au-based D and S electrodes on a SiO_2_/Si wafer. Scheme 1 shows schematic diagrams of (a) the bioactivation of MWCNTs with MCTA through an imide formation reaction between H_2_N– of the MCTA and HOOC– of the MWCNTs, and (b) the selective interactions between MC-LR and MCTAs. Bovine serum albumin (BSA) was coated on the MWCNT surfaces (Scheme 1a) to prevent the random adsorption of undesired chemicals and avoid electrical conduction between the MWCNT filaments owing to its insulating characteristics. Therefore, the entanglement between the *p*-type MCTA-MWCNTs is the putative electrical connection route. The primary detection mechanism is the untangling of intercrossed MCTAs upon the selective capture of MC-LR, as shown in Scheme 1b. The targeted MC-LR could be captured only at the activated site (–MCTA) via a lock-and-key mechanism of the bio-selective reaction, which led to an increase in the potential barrier in the electrical conduction path between the MWCNTs, and a consequent increase in electrical resistivity (*ρ*). The change in *ρ* is an essential mechanism for detecting MC-LR using these bioactivated MWCNT [29]. The change in the potential barrier between MCTAs will be more sensitive in the top-gated FET configuration than in the compressed bulk of the MCTA-MWCNTs, which is the key design principle of this study. In the FET configuration, the response signals can be readily enhanced by applying a higher gate voltage [30]. This method can be applied for the high-speed and rapid (<10 min) detection of MC-LR at any outbreak location.

## 2. Materials and Methods

### 2.1. Materials

Nitric acid (64.0–65.0%) was purchased from Duksan Science (Ansan, Republic of Korea). Phosphate-buffered saline (PBS, pH 7.2) was purchased from Biosesang Inc. (Seongnam, Republic of Korea). 2-(*N*-Morpholino)ethanesulfonic acid (MES, 1 M, pH = 5.0) and a solution of 0.05% Tween 20 + 1% BSA in PBS were purchased from Tech & Innovation (Chuncheon, Republic of Korea). *N*-(3-Dimethylaminopropyl)-*N*-ethylcarbodiimide hydrochloride (EDC) was purchased from Sigma-Aldrich (St. Louis, MO, USA). MC-LR, MC-LY (L: leucine, Y: tyrosine), and MC-YR (Y: tyrosine, R: arginine) were purchased from Enzo Life Sciences, Inc. (Farmingdale, NY, USA). The MC-LR-targeted synthetic DNA (MCTA; DNA oligonucleotide 5-NH_2_-C_6_-AN_6_) was prepared by Macrogen (Seoul, Republic of Korea). Micropore filter paper (mixed cellulose ester, Ø25 mm, 0.45 µm-sized pores) was purchased from Toyo Roshi Kaisha, Ltd. (Tokyo, Japan). MWCNTs with a diameter of 20 nm and a length of 5 µm were purchased from Carbon Nano-Material Tech. Co. (Pohang, Republic of Korea). All chemicals were used as received without further purification for the bioactivation procedure. 

### 2.2. Functionalizaiton of Multi-Walled Carbon Nanotubes

**Figure 1:**
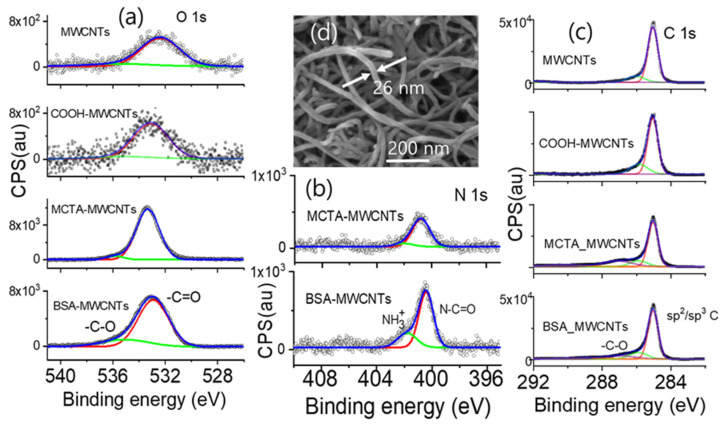
X-ray photoelectron spectroscopy (XPS) profiles near binding energy of (**a**) oxygen, (**b**) nitrogen, and (**c**) carbon for the functionalized and pristine MWCNTs. High-resolution N 1s spectra for MWCNTs and COOH-MWCNTs were not obtained, because of the lack of detectable signals in the survey spectrum. (**d**) Scanning electron microscopy (SEM) image of the MCTA-MWCNT filaments with a diameter of ~26 nm, close to that of the pristine MWCNTs.

The bioactivation of the MWCNTs was conducted following a previously reported method [29]. The MWCNTs were carboxylated by *c*-HNO_3_ treatment, as shown in Scheme 1, in which MWCNTs (300 mg) were reacted with HNO_3_ (150 mL, 3 M) for seven days at 130 °C, and subsequently washed via more than ten cycles of centrifugation to achieve a pH of approximately 7. MCTA was immobilized on the MWCNTs by the formation of an amide group via the reaction of a mixture containing carboxylated MWCNTs (5.2 mg), N-(3-dimethylaminopropyl)-N-ethylcarbodiimide hydrochloride (EDC; 4 μL), and MC-LR-targeting aptamers (MCTAs; 2 mL, 66 nM) in 16 mL of 0.1 M MES for 24 h. The MWCNTs were coated with BSA to prevent undesired interactions between the surfaces of the MWCNTs and analytes or interfering agents. The samples prepared at this stage were denoted as MCTA-MWCNTs to indicate the attachment of MCTAs to the MWCNTs. After each step of the preparation procedure, the modified MWCNTs were analyzed using Fourier transform infrared spectrometry (FT-IR; JASCO FT/IR-4100, Easton, MD, USA). The chemical environment of the MCTA-attached MWCNTs was confirmed by X-ray photoelectron spectroscopy (XPS; Multilab2000, Thermo Scientific, Waltham, MA, USA), as shown in Figure 1. The XPS profiles of the samples were deconvoluted to confirm the bioactivation, which was analyzed primarily based on the FT-IR spectra in our previous study [29].

### 2.3. Fabrication of MWCNT-Based Top-Gate FET Device

The MCTA-MWCNTs were stored in water (0.5 mg/mL) and diluted to 0.05 mg/mL prior to the FET assembly. Interdigitated Au electrodes (DH gate—Hxq315, Guangdong, China; 150 nm thick Au layer on 10 nm thick Cr layer on SiO_2_/Si) with a 20 μm wide gap were used as the D and S electrodes. The MWCNTs were examined using scanning electron microscopy (SEM, Hitachi S2400, Tokyo, Japan). A Ag/AgCl electrode was used as the top-gate (G) electrode. The FET was assembled using a probe station by controlling the 1 μm scale with the *XYZ* stage. Interdigitated Au electrode was prepared as follows. The Au electrode was cleaned with a Au cleaning solution (Sigma-Aldrich, St. Louis, MO, USA) for 5 s, washed with distilled water, and dried with pure N_2_ (99.99%, Hanagas, Gimhae, Republic of Korea). One drop (1 μL) of the MCTA-MWCNT suspension (0.05 mg/mL) was placed on the cleaned Au electrodes and subsequently dried in a convection oven at 50 °C for 30 min to immobilize the MCTA-MWCNTs between the D and S electrode fingers (Figure S1). A rectangular well (2 × 4 × 2 mm; 16 μL) made of polydimethylsiloxane (PDMS; Dow Corning Sylgard 184, Midland, MI, USA) was used as the solution container and positioned on an individual Au electrode. The G electrode was immersed in the test solution to configure the top-gated FET. These FETs offer advantages in terms of requiring a significantly smaller gate voltage range (*V*_G_ ~ ±0.5 V) compared to that of the back-gating operation (±20 V) [31,32]. In addition, the sensitivity of top-gated FET sensors is typically ten times higher than that of back-gated FET sensors. The fabricated FET sensor maintained its detection characteristics for at least 60 days.

### 2.4. Characterization and Measurement of the Device

The MC-LR solutions with varying concentrations, ranging from 0 to 1.0 ng/mL were prepared in 1 × phosphate-buffered saline (PBS). Sixteen microliters of the MC-LR solution was loaded into a PDMS well on the Au electrode. Subsequently, the *I*_ds_-*V*_g_ characteristics with a constant *V*_ds_ of −0.5 V and *I*_ds_-*V*_ds_ characteristics with *V*_g_ varying from 0 to −1 V were instantaneously measured using a computer interface connected to a constant-current source (digital multimeter; Keithley 196, Cleveland, OH, USA) and a DC power source (T7 with LJTick-DAC, Labjack, Lakewood, CO, USA). After each measurement, the Au electrode was replaced with another subsequent concentration. In this study, the Au electrodes were reused three times after rinsing with the cleaning solution. Unless otherwise specified, all measurements were performed at least in triplicate at room temperature. The limit of detection (LOD) of the device was determined as follows: LOD = 3.3 × SD of intercept/|slope| from the g_m_/g_mo_ vs. MC-LR concentration plot, where SD is standard deviation.

To validate the performance of the sensor for real samples, we collected environmental waters in sterile glass bottles in October 2021 from two dams, the Yeongju Dam (YD, 36°72′ N 128°66′ E) and the Andong Dam (AD, 36°58′ N 128°77′ E), located upstream of the Nakdong River (Republic of Korea). Algal blooms were severe in the YD over eight years until late autumn owing to eutrophication, mainly caused by anthropogenic activities from industrial and agricultural complexes [33]. However, algal blooms in AD usually decreased or disappeared during autumn. All water samples were filtered using a microfilter paper with 0.45 μm sized pores. The assay was completed within 10 min. The MC-LR levels in freshwater were assayed using LC-MS/MS (Waters XEVO TQ-S Micro, Milford, MA, USA) to confirm the performance of the fabricated FET biosensor. The water components were quantified using the Korean standard procedure for drinking water analysis [34]. The electrical characteristics of the FET were evaluated using an electrometer (Keithley K617, Cleveland, OH, USA), digital voltmeter (Keithley K2182, Cleveland, OH, USA), and digital I/O board (LabJack U12, Lakewood, CO, USA) with a personal computer for data acquisition. A printed circuit board and universal-serial-bus-controllable multifunction digital acquisition board (LabJack T7) can be used as replacement for the probe station and other instruments.

## 3. Results and Discussion

### 3.1. Fabrication of the FET Sensor

The bioactivation of the MWCNTs was confirmed by XPS analysis, as shown in Figure 1. In Figure 1a, the O 1s band of the MWCNTs is significantly weak, which is owed to adventitious carbon contamination. Upon carboxylation (COOH-MWCNTs), the O 1s band shifts slightly to a higher binding energy. The intensity of the O 1s band was significantly higher for the MCTA-MWCNTs and BSA-coated MCTA-MWCNTs because of the presence of oxygen atoms in the MCTAs and BSAs. As shown in Figure 1b, the N 1s band of these samples originated from MCTAs and BSA. The N 1s band position (400 eV) corresponds to N in the amide (O=C–N–) group, and the shoulder peak (~402 eV) corresponds to protonated N in the amide group N [35]. In Figure 1c, the band position of the shoulder peak of C 1s, which corresponds to the oxygenated carbon species, was enhanced and shifted to a higher energy by bioactivation. These results confirm bioactivation and agree with the FT-IR analysis described previously [29]. Figure 1d shows the SEM image of the bioactivated MWCNTs. The diameter of the MCTA-MWCNTs was similar to that of the pristine MWCNTs.

**Figure 2:**
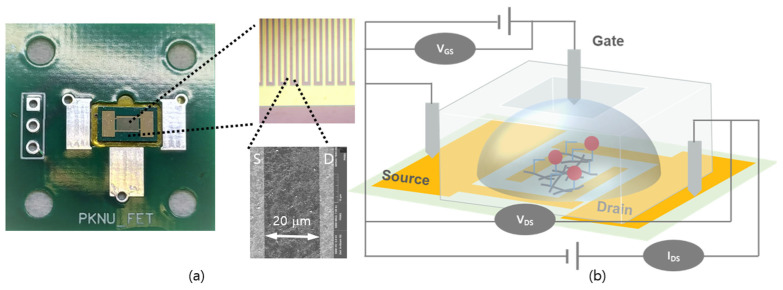
(**a**) Optical image of the drain (D) and source (S) Au electrodes of the field-effect transistor (FET) sensor. The magnified SEM image reveals a gap of 20 µm between D and S. (**b**) Schematic diagram of the top-gated FET fabricated in this study. In this figure, the electrode was simplified for better visualization.

Fabrication of the bioactivated MWCNT-based FET sensor is illustrated in Figure 2. Figure 2a shows an optical image of the D and S electrodes situated on a SiO_2_/Si wafer, comprising 60 interdigitated fingers. The magnified SEM image in Figure 2a shows the MCTA-MWCNTs immobilized randomly between the D and S electrode fingers. Figure 2b shows a schematic diagram of the MWCNT-FET. After immobilization of the MCTA-MWCNTs, the electrical resistance (*R*) between the D and S electrodes decreased from >40 MΩ to ~4 KΩ. The *R* value after a subsequent wash with PBS was not significantly altered (<3%). This indicated that the spatial arrangement of the immobilized MCTA-MWCNTs remained unaltered after washing (Figure S1). The Au cleaning procedure facilitated the activation of the Au surface by removing adsorbed organic chemicals. The cleaned Au surface demonstrated increased reactivity with the organic functional moieties of the activated MWCNTs, such as –OH, –COOH, and –NH_2_ [36]. Within two hours of the self-assembly reaction under ambient laboratory conditions, the contact angle of a water droplet on the Au surface increased from <5° to ~60° [36]. These data indicate that the cleaned Au surface was hydrophilic, but was altered to a hydrophobic surface by the deposition of MWCNTs. Notably, immersion in a Au cleaning solution or gentle wiping using a cotton swab can detach the immobilized MCTA-MWCNTs. Therefore, meticulous handling is imperative to ensure consistent fabrication of the sensor.

### 3.2. Characteristics of Device Performance

**Figure 3:**
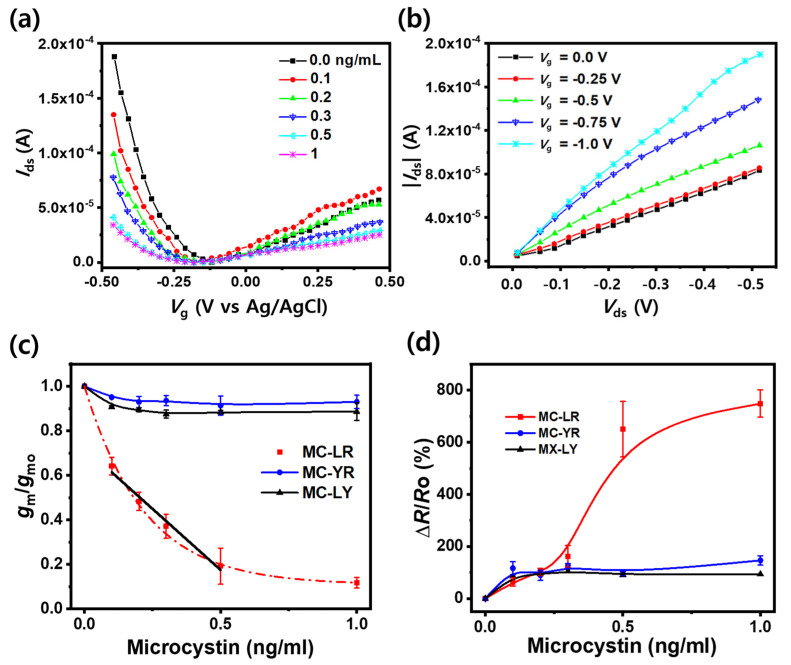
(**a**) Ambipolar characteristic of the MCTA-MWCNT FET at a constant drain–source potential (*V*_ds_ = −0.5 V) with varying concentrations of MC-LR in PBS buffer. (**b**) Negative gate-potential-controlled *I*–*V* characteristic between the drain/source electrodes of the FET obtained using 0.1 ng/mL of MC-LR in PBS buffer. (**c**) Relative transconductance (g_m_/g_mo_) of the FET for MC congeners. The black line is the linear regression of values from 0.1, 0.2, 0.3, 0.5 ng/mL of MC-LR. (**d**) Sensitivity (Δ*R*/*R*o) of the FET for MC congeners. The line through the MC-LR data in (**c**) represents the fit described in Results 3.3.

We evaluated the performance of the FET sensor in detecting MC-LR. Figure 3a shows plots of the D-S current (*I*_ds_) as a function of the gate voltage (*V*_g_) of the fabricated MWCNT-FET recorded at a constant *V*_ds_ (−0.5 V) across varying concentrations of the MCTAs (0–1 ng/mL) in PBS buffer. The plot highlights the ambipolar electric-field effect of the FET, with the lower and higher *V*_g_ sides of the Dirac point (lowest *I*_ds_ value) exhibiting *p*- and *n*-characteristics, respectively. Such an ambipolar conductance has frequently been observed in liquid gate (top-gate) FETs [32]. As the MC-LR concentration increased, the magnitude of the slope decreased in both regions. This trend suggests that the resistivity (*ρ*) of the MCTA-MWCNTs increases upon MC-LR binding. The preferential affinity between MC-LR and MCTAs was markedly stronger than that between the non-specific interactions among MCTAs. Hence, MCTAs predominantly capture MC-LR rather than remaining in the entangled state. The capturing event causes gradual loosening of the MCTA entanglement, which increases the potential barrier for conduction and, in turn, *R* of the FET. Moreover, the increment in *ρ* results in a decrease in the magnitude of the slope of the *I*_ds_ vs. *V*_G_ profile. Figure 3a reveals that the *p*-characteristic region of the MWCNT-FET was more sensitive than the *n*-characteristic region when the FET sensor was exposed to an MC-LR-spiked PBS solution. Notably, the Dirac point, positioned at −0.17 V to the FET in pure PBS buffer, shifted to a more negative value (−0.22 V) in the MC-LR-spiked PBS solution (≥0.1 ng/mL). The fabricated MCTA-MWCNT FET is an *n*-doped *p*-type FET [37,38,39,40,41,42,43,44,45]. The left shift of the Dirac point from −0.17 V to −0.22 V implied that more electrons were doped into MWCNTs when MC-LR, which is weakly and negatively charged at pH 7.2, was bonded to the MC-TA aptamer. The working principle of this FET can be explained by charge transfer from either the MC-LR or MC-LR-MCTA aptamer and not by the electrostatic gating effect [23,38,42,44,45,46,47]. Consistent with other observations [48,49], the *n*-characteristic region is less defined probably because of the adsorption of oxygen in the solution. Although a quasi-linear correlation exists between *I*_ds_ and the MC-LR level at a fixed *V*_g_ in the *n*-type region (Figure 3a), this relationship is less defined in the dilute MC-LR concentration region (<0.2 ng/mL). Therefore, the electrical characteristics of the *p*-type region were adopted as the sensing signals for the FET sensor.

In contrast, Figure 3b delineates the relationship between *I*_ds_ and *V*_ds_ of the FET exposed to 0.1 ng/mL of MC-LR, with *V*_g_ ranging from 0 V to −1 V. In Figure 3b, the gated effect was discernibly evident at *V*_g_ = −0.5 V, with the negative value implying that the active element (MCTA-MWCNTs) in the MWCNT-FET manifests *p*-type conduction characteristics [32]. 

### 3.3. Sensitivity and Selectivity

The sensing performance of the FET is typically determined by its transconductance, which is denoted by *g*_m_ [26,27]. This parameter, *g*_m_, is defined as the slope of the *I*_ds_ vs. *V*_g_ plot at a constant *V*_ds_, expressed as *g*_m_ = Δ*I*_ds_/Δ*V*_g_. We obtained *g*_m_ from the *p*-characteristic region of Figure 3a, since the response in the *p*-type region was more stable and sensitive. In this region, the dependency of *g*_m_ on the hole mobility, *μ_h_*, is governed by the equation, *g*_m_ = *μ_h_* (*C*/*L*^2^)*V*_ds_, where *C* represents the capacitance of the device and *L* is the length of the conductor [26,27]. Considering the values of *C*, *L*, and *V*_ds_ remain constant for the FET, *g*_m_ is a function of *μ_h_*. This relationship suggests that the ratio *g*_m_/*g*_mo_, representing the relative transconductance of the MC-LR-dosed FET device (where *g*_mo_ denotes the transconductance, *g*_m_, of the background PBS solution), can serve as the sensing signal of the sensor. The relative hole mobility of the sensing component in the FET sensor, represented by *μ_h_*/*μ*_o*h*_ (where *μ*_o*h*_ signifies the *μ_h_* for PBS solution), undergoes a decline owing to the enhanced potential barrier amid the MCTA-MWCNTs upon capturing MC-LR by the MCTAs, as previously discussed. However, the electrical-field effects (shift in Dirac point, increase in *ρ*, and decrease in relative transconductance with an increase in MC-LR concentration) were not observed in the BSA-MWCNT-FET (FET assembled with BSA-coated pristine MWCNTs). Such inactive FET signals (Figure S2) indicated the absence of bioactive sites in the BSA-coated MWCNTs.

Figure 3c delineates the *g*_m_/*g*_mo_ values of the FET as a function of the spike level of MC congeners (MC-LR, MC-YR, and MC-LY) in PBS solution at fixed *V*_ds_ = −0.5 V and *V*_g_ = −0.5 V. MC-LR, MC-YR, and MC-LY are commonly found MC congeners in natural freshwater [4]. The *g*_m_/*g*_mo_ for MC-LR was deduced from the *p*-characteristic side of the Dirac point, as shown in Figure 3a. Additionally, the *g*_m_/*g*_mo_ values of MC-YR and MC-LY were estimated from the corresponding *I*_ds_ vs. *V*_g_ plots (Figure S3). The *g*_m_/*g*_mo_ curve for MC-LR, which represents the sensing signal, was fitted using an exponential function for the data in the range of 0 to 1 ng/mL, *y* = *A*_1_ exp(−*x*/*t*_1_) + *y*_o_, where *A*_1_ = 0.891, *y*_o_ = 0.109, and *t*_1_ = 0.219 (with *R*^2^ = 0.98). A linear relationship can be established at the low concentrations of 0.1, 0.2, 0.3, and 0.5 ng/mL of MC-LR (Figure 3c), with the linear regression characterized by slope, intercept, and *R*^2^ values of −1.10, 0.0374, and 0.97, respectively (Table S1). The initial data point (*x* = 0 ng/mL) was excluded for the optimal fit. Based on this linear model, the sensor’s detection limit of the sensor was estimated to be 0.11 ng/mL. The performance of the MCTA-MWCNT-FET sensor fabricated in this study was compared with other electrochemical sensors with high sensitivity (Table 1). The proposed FET sensor has a narrower detection range and a higher detection limit than most other sensors except for the MWCNT and SWCNT immunosensors [50,51]. This result is supported by the fact that graphene- and SWCNT-based sensors are, generally, more sensitive than MWCNT-based sensors [52,53,54]. However, the detection limit of the proposed FET sensor was low for practical use.

Figure 3d illustrates the selectivity of the FET sensor, represented as Δ*R*/*R*_o_ × 100, in response to varying concentrations of MC-LR, MC-YR, and MC-LY. Despite the substantial structural similarities among MC-LR, MC-YR, and MC-LY, the MCTA-MWCNT FET sensor demonstrated a pronounced selectivity toward MC-LR in the examined concentration range. In the concentration range of 0.0–1.0 ng/mL, MC-YR and MC-LY exhibited nearly invariant and relatively small values of the *g*_m_/*g*_mo_, whereas *g*_m_/*g*_mo_ decreased consistently with increasing MC-LR concentration. This observation indicates the selectivity of the fabricated FET sensor for MC-LR in the presence of its congeners.

**Figure 4:**
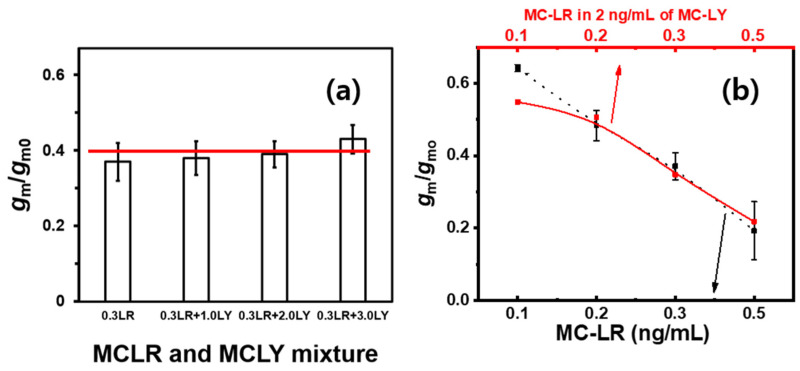
Relative transconductance values of (**a**) MC-LR (0.3 ng/mL) estimated at high levels of MC-LY-spiked PBS buffer (1.0, 2.0, and 3.0 ng/mL), the red line is the relative transconductance of 0.3 ng/mL MC-LR, and (**b**) MC-LR (0.1–0.5 ng/mL) at a high concentration of MC-LY-spiked PBS buffer (2.0 ng/mL).

Thus, the selectivity of the MC-LR sensor in the presence of potential interferents was corroborated. Figure 4a shows the *g*_m_/*g*_mo_ values corresponding to the PBS solutions spiked at concentrations of 0, 1.0, 2.0, and 3.0 ng/mL of MC-LY against a constant MC-LR concentration of 0.3 ng/mL. The observed *g*_m_/*g*_mo_ values indicated that the FET signal remained unaffected within the error range even when the MC-LY concentration surpassed that of MC-LR by a factor of ten. Figure 4b shows the selective detection capability of the FET sensor for various MC-LR concentrations at a constant MC-LY concentration of 2.0 ng/mL. The black dotted line in the plot, symbolizing the FET sensing signal for MC-LR in PBS buffer, presents a linear relationship between *g*_m_/*g*_mo_ and the MC-LR concentration, as shown in Figure 3a. Remarkably, both profiles coincided over almost the entire concentration range, except for <0.2 ng/mL of MC-LR. These data confirmed the low selectivity of the FET sensor at low concentrations. The selectivity of the sensor was observed at other concentrations of MC-YR and MC-LY (Figures S3 and S4). However, the red profile represents the FET-sensing signal for MC-LR in 2.0 ng/mL MC-LY in PBS.

### 3.4. Matrix Effect and Actual Application

**Figure 5:**
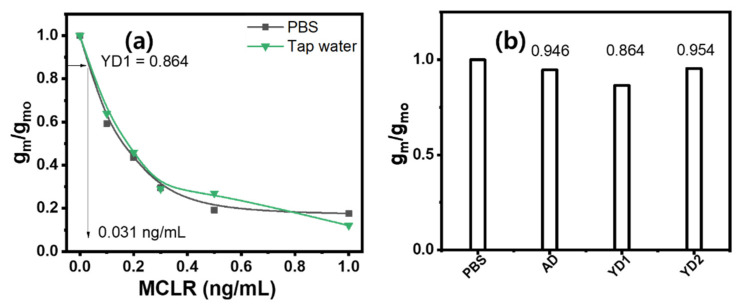
(**a**) Relative transconductance of the FET sensor for tap water and MC-LR in PBS buffer. (**b**) Detection signals of the MCTA-MWCNT FET for PBS buffer, and Andong dam (AD) and Yeongju dam (YD1 and YD2) water samples.

Biochemical assays of natural waters can be affected by diverse chemical and physical contaminants present in the water [58]. Thus, it is imperative to carefully examine the potential matrix effects, especially when the assay is geared toward detecting trace levels of chemical constituents in natural water resources. Notably, the MCTA-MWCNT-based FET displayed a conspicuous absence of the matrix effect. Figure 5a indicates that the *g*_m_/*g*_mo_ values corresponding to 0, 0.2, 0.3, 0.5, and 1.0 ng/mL of MC-LR-spiked tap water filtered with 0.45 μm sized pores aligned with those from equivalent MC-LR levels in PBS. This observation suggests that the calibration curve obtained using PBS buffer as a surrogate for tap water sourced from the Nakdong River (Busan, Republic of Korea) remains valid for assessing MC-LR levels in water from this river.

Water samples from the two reservoirs were collected to represent conditions with and without algal blooms. The samples collected from the Yeongju Dam (designated YD1 and YD2) displayed discernible greenish particulate matter, indicative of an algal bloom. However, samples from the Andong Dam (designated AD) presented no evident particulate debris. Optical images of the residues on the filter paper revealed differences between the water samples from the two dams (Figure S5). The two filters with green residues correspond to the YD water samples. The color observed in the UV–vis spectrum, as presented in Figure S5, is consistent with the known absorption properties of chlorophyll A and B [59].

The LC-MS/MS analysis revealed that the concentration of MC-LR in both water samples was below the detection limit of the instrument (0.01 ng/mL). This observation may be odd because the two samples, YD1 and 2, were obtained from moderately algal blooming water bodies. We speculate that microcystins are intracellular toxins that are usually released when cyanobacteria are lysed or old [60]. However, the reading of the fabricated FET sensor indicated that the MC-LR concentration in the sample was <0.031 ng/mL, as shown in Figure 5. This false-positive reading can be attributed to the MC-LR concentration residing below the error range of the detection limit. Although the ultimate detection limit of the fabricated FET sensor does not match that of the LC-MS/MS method, the sensor can be applied for the rapid preliminary evaluation of environmental water at point sources. Consequently, the designed bioactivated MWCNT-FET sensor is a time-saving, convenient, inexpensive, and reliable environmental sensor for detecting MC-LR in freshwater systems.

## 4. Conclusions

A bioactivated multiwalled carbon nanotube (MWCNT)-based label-free field-effect transistor (FET) sensor was developed to detect microcystin-LR (MC-LR). The active element of the FET was MWCNTs activated with an MC-LR-targeting aptamer (MCTA). This sensor exhibits high sensitivity, excellent selectivity even in the presence of other MC congeners, and no matrix effects even in the presence of other MC congeners. This environmental FET sensor was found to assay the MC-LR level in the range of 0.1–0.5 ng/mL, with a detection limit of 0.11 ng/mL, within 10 min. This methodology holds promise for the expeditious and sensitive detection of MC-LR in algal bloom locations in freshwater systems.

## Figures and Tables

**Table 1:** Comparison of analytical performance of the MCTA-MWCNT with other electrochemical sensors for detection of microcystin-LR ^1^.

Sensors	Detection Techniques	Linear Ranges (µg/L)	LOD	References
MWCNTs/Ab	CV, EIS	0.05–20	0.04 µg/L	[50]
SWCNTs/Apt	Fluorometry	-	0.134 pM	[55]
MWCNTs/Ab	CV, EIS	0.005–1.0	1.7 ng/L	[14]
Nanobiochar/Ab	Amperometry	0.1–100	0.017 µg/L	[37]
CNx-MWNTs/Ab	DPV	0.01–2.00	0.004 µg/L	[56]
SWCNTs/Ab	Electrical resistance	0.001–1.00	0.6 ng/L	[8]
AuNP/Graphene SERS-FET/Ab	SERS-FET	1 × 10^−8^–10^−18^ M	0.62 aM	[57]
SWCNTs/Ab	Amperometry	10.00–40.00	0.6 µg/L	[51]
Graphene/Apt	SWV	0.001–1.00	1.9 ng/L	[10]
MWCNTs/Apt	FET	0.1–0.5	0.11 µg/L	In this study

^1^ Abbreviations: Ab, antibody; Apt, aptamer; CNx-MWNTs: nitrogen-doped multi-walled carbon nanotubes; CV, cyclic voltammetry; DPV, differential pulse voltammetry; EIS, electrochemical impedance spectroscopy; SERS, surface-enhanced Raman scattering; SWV, square wave voltammetry.

## Data Availability

Data are contained within the article.

## Supplementary Materials

The following supporting information can be downloaded at https://www.mdpi.com/article/10.3390/bios14010037/s1, Figure S1. SEM images of the (a) MCTA-MWCNTs immobilized between the drain and source electrodes by solution dropping, and immobilized MCTA-MWCNTs after washing with PBS (scale bars: 10 μm), and (b) MCTA-MWCNTs entangled on the surface of the electrode. Figure S2. *I*_DS_–*V*_G_ plots of the FET assembled using BSA-coated MWCNTs at a constant drain–source potential (*V*_DS_ = −0.5 V) with varying concentrations of MC-LR in PBS buffer, confirming the lack of bioactivity of the MWCNT-FET sensor without the MCTAs. The relative transconductance values (*g*_m_/*g*_mo_) corresponding to the various levels of MC-LR are identical. Figure S3. *I*_DS_–*V*_G_ characteristic curves of the MCTA-MWCNT FET corresponding to various concentrations of (a) MC-YR and (b) MC-LY in PBS buffer, at a *V*_DS_ of −0.5 V. Figure S4. Relative conductance of MC-LR (0.3 ng/mL) with respect to various combinations of the MC-LY and MC-YR levels. In the *x*-axis, LR03, YR1, and LY1 correspond to MC-LR (0.3 ng/mL), MC-YR (1.0 ng/mL), and MC-LY (1.0 ng/mL) in PBS buffer. Figure S5. (a) Optical images of water sourced from the Yeongju dam (YD1 and YD2) and Andong dam (AD), before and after filtering; the used filter papers are also shown. (b) UV–vis spectrum of YD1.
