# Supplementary material for: Rapid and Easy Detection of Microcystin-LR Using a Bioactivated Multi-Walled Carbon Nanotube-Based Field-Effect Transistor Sensor
Source: Biosensors (Basel). 2024 Jan 11;14(1):37. doi: 10.3390/bios14010037 (PMC10813581; doi:10.3390/bios14010037)
Supplement: Supplementary file 1 [file biosensors-14-00037-s001.zip › biosensors-2767545-supplementary.pdf]

Supplementary Materisls

Rapid and easy detection of microcystin-LR using a bioactivated multi-walled carbon nanotube-based field-effect transistor sensor

Myeongsoon Lee [1], Seong H. Kim [2], Don Kim [1] and Hak Jun Kim [1,*]

[1] Department of Chemistry, Pukyong National University, Busan 48513, Republic of Korea; kimhj@pknu.ac.kr
[2] Department of Chemical Engineering and Materials Research Institute, Pennsylvania State University, University Park, PA 16802, USA; shk10@psu.edu

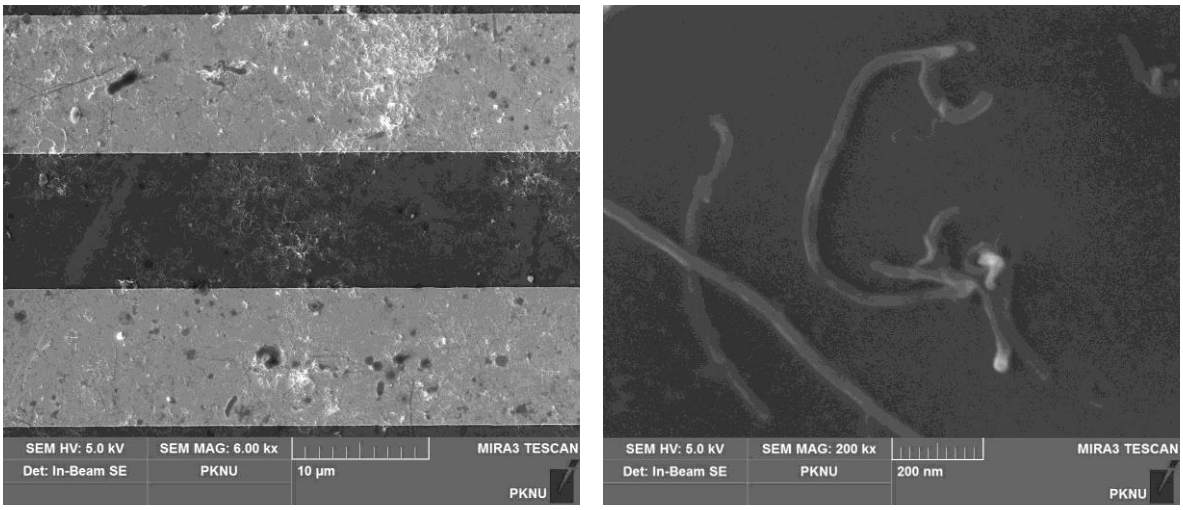

(a) (b)

Figure S1. SEM images of the (a) MCTA-MWCNTs immobilized between the drain and source electrodes by solution dropping, and immobilized MCTA-MWCNTs after washing with PBS (scale bars: 10 μm) and (b) MCTA-MWCNTs entangled on the surface of the electrode.

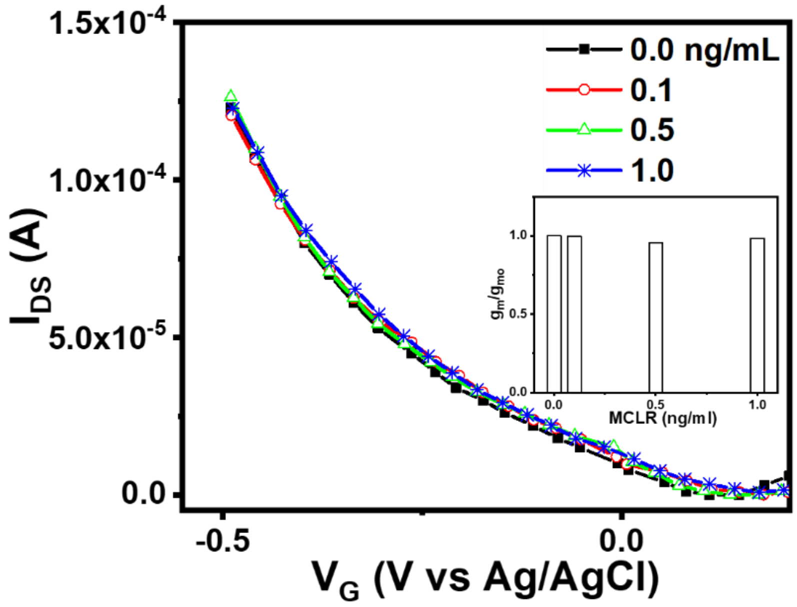

Figure S2. I_{DS}–V_G plots of the FET assembled using BSA-coated MWCNTs at a constant drain-source potential (V_{DS} = −0.5 V) with varying concentrations of MC-LR in PBS buffer, confirming the lack of bioactivity of the MWCNT-FET sensor without the MCTAs. The relative transconduction values (g_m/g_{mo}) corresponding to the various levels of MC-LR are identical.

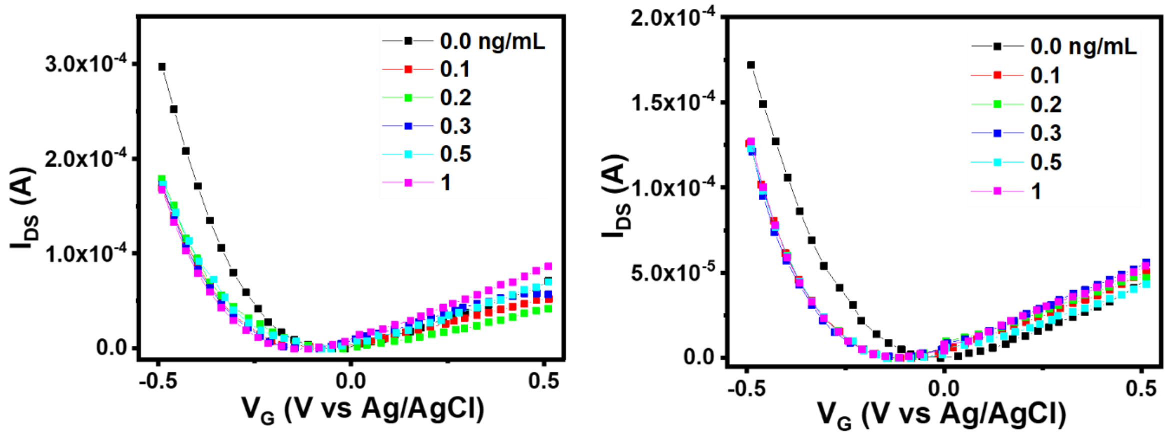

Figure S3. I_{DS}–V_G characteristic curves of the MCTA-MWCNT FET corresponding to various concentrations of (a) MC-YR and (b) MC-LY in PBS buffer, at a V_{DS} of −0.5 V.

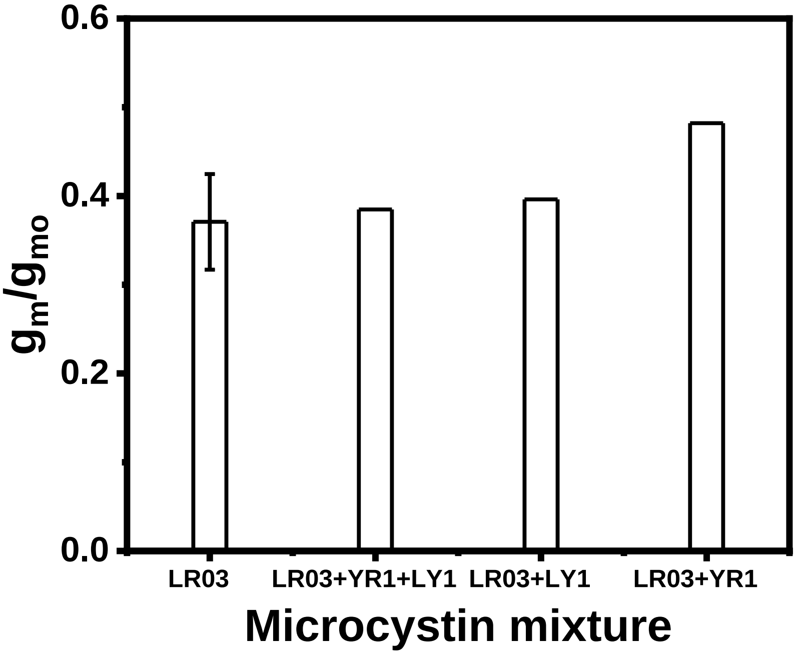

Figure S4. Relative conductance of MC-LR (0.3 ng/mL) with respect to various combinations of the MC-LY and MC-YR levels. In the *x*-axis, LR03, YR1, and LY1 correspond to MC-LR (0.3 ng/mL), MC-YR (1.0 ng/mL), and MC-LY (1.0 ng/mL) in PBS buffer, respectively.

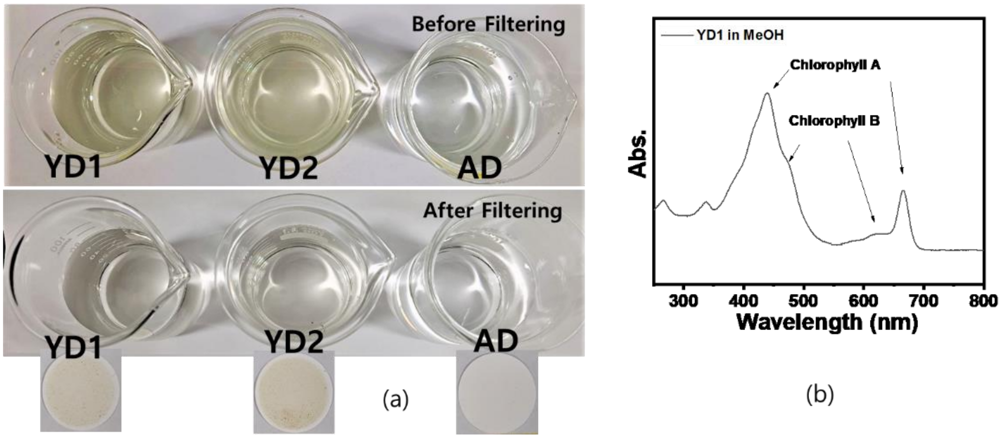

Figure S5. (a) Optical images of water sourced from the Yeongju dam (YD1 and YD2) and Andong dam (AD), before and after filtering; the used filter papers are also shown. (b) UV–vis spectrum of YD1.
